# Biological variation of serum insulin concentrations in healthy cats

**DOI:** 10.1186/1751-0147-57-S1-O14

**Published:** 2015-09-25

**Authors:** Emma Strage

**Affiliations:** 1 Department of Clinical Sciences, Swedish University of Agricultural Sciences, Uppsala, Sweden; 2 Clinical Pathology Laboratory, University Animal Hospital, Swedish University of Agricultural Sciences, Uppsala, Sweden

## Introduction

The prevalence of diabetes mellitus (DM) in cats is increasing. It has been proposed that fasting concentrations of insulin and glucose can be used for screening of reduced insulin sensitivity in cats. Reduced insulin sensitivity may indicate that cats are at risk of developing DM and motivate early preventive actions such as weight reduction and change of diet. To correctly interpret results it is important to know the biological variation of insulin i.e. the normal variation of insulin between and within cats. For glucose, coefficient of variation (CV) for between and within cats is reported to be 8.1 and 6.8%, respectively, but until now there are no data of biological variation of insulin in cats.

## Objectives

To determine biological variation of insulin in healthy cats.

## Methods

Healthy cats (n=5) were sampled in their home environment once a week for 5 weeks. Blood samples were drawn from the cephalic vein in the morning after >12 hour fast. Insulin was measured in duplicate by the previously validated Mercodia feline insulin ELISA. Nested analysis of variance was used for statistical calculations.

## Results

Insulin ranged from 71.8-304.5 (median 127.3) ng/L. CV between and within cats was 58.0% and 34.1%, respectively.

## Conclusions

Biological variation of insulin within a cat is considerably higher than previously published results for glucose. This needs to be taken into account when interpreting insulin concentrations.

